# Mycoalgae biofilm: development of a novel platform technology using algae and fungal cultures

**DOI:** 10.1186/s13068-016-0533-y

**Published:** 2016-05-31

**Authors:** Aravindan Rajendran, Bo Hu

**Affiliations:** Department of Bioproducts and Biosystems Engineering, University of Minnesota, Room: 219, BioAgEng, 1390 Eckles Ave, St. Paul, MN 55108-6005 USA; Department of Bioproducts and Biosystems Engineering, University of Minnesota, Room: 315, 1390 Eckles Ave, Saint Paul, MN 55108-6005 USA

**Keywords:** *Mycoalgae* biofilm, Filamentous fungi, Microalgae, Co-culture, Bioprocessing, Kinetics

## Abstract

**Background:**

Microalgae is considered a promising source for biofuel and bioenergy production, bio-remediation and production of high-value bioactive compounds, but harvesting microalgae is a major bottleneck in the algae based processes. The objective of this research is to mimic the growth of natural lichen and develop a novel biofilm platform technology using filamentous fungi and microalgae to form a lichen type of biofilm “mycoalgae” in a supporting polymer matrix.

**Results:**

The possibility of co-existence of *Chlorella vulgaris* with various fungal cultures was tested to identify the best strain combination for high algae harvest efficiency. The effect of different matrices for cell attachment and biofilm formation, cell surface characterization of mycoalgae biofilm, kinetics of the process with respect to the algae-fungi cell distribution and total biomass production was studied. Mycoalgae biofilm with algae attachment efficiency of 99.0 % and above was achieved in a polymer-cotton composite matrix with glucose concentration of 2 g/L in the growth medium and agitation intensity of 150 rpm at 27 °C. The total biomass in the co-culture with the selected strain combination (*Mucor* sp. and *Chlorella* sp.) was higher than the axenic cultures of fungi and algae at the conditions tested.

**Conclusions:**

The results show that algae can be grown with complete attachment to a bio-augmenting fungal surface and can be harvested readily as a biofilm for product extraction from biomass. Even though, interaction between heterotrophic fungi and phototrophic algae was investigated in solid media after prolonged contact in a report, this research is the first of its kind in developing an artificial lichen type biofilm called “mycoalgae” biofilm completely attached on a matrix in liquid cultures. The mycoalgae biofilm based processes, propounds the scope for exploring new avenues in the bio-production industry and bioremediation.

**Electronic supplementary material:**

The online version of this article (doi:10.1186/s13068-016-0533-y) contains supplementary material, which is available to authorized users.

## Background

In natural ecosystems, most microbes exist as part of a complex, dynamically changing, microbial consortia, and the metabolic interactions between microbial species could be for various reasons including, exchange of molecules, which may benefit one or both species [1]. There are many reports suggesting the benefits of co-cultures in a synthetic ecosystem for a variety of applications; specifically to perform functions requiring multiple steps, including bio-remediation, and for organisms that are often difficult to manipulate genetically [1]. In the context of co-existence, fungi exhibit efficient symbiotic capabilities with high ecological significance playing a vital role in ecosystem function and the maintenance of biodiversity. Lichens are one of the most significant and widely witnessed fungal symbiotic relationships, and more than one-fifth of all extant fungal species are known to be lichenized [2]. Lichens are the symbiotic phenotype of nutritionally specialized fungi that live as ecologically obligate biotrophs in symbiosis with photoautotrophic green algae, cyanobacteria, or both types of photobionts. Natural lichen systems, despite their high ecological importance, seldom have any industrial applications because their growth is too slow [2]. But, they have successfully adapted to life under harsh conditions and therefore can provide a unique view to improve current industrial systems. Less is known about the physiological aspects of lichens than those of other symbiotic systems, such as the rhizobium-legume relationship or endophytic fungi.

Even though microalgae has the potential to become a substantial driver in the development of a bio-based economy, large-scale applications of microalgae biotechnology are hampered by the current energy-intensive or non-ecofriendly chemical harvest technology [3], along with the high nutrient and water requirements. Also, the success of the large scale open ponds for algae production is challenged by the invasion of local biota. Due to the small cell size of algae with specific gravities being very similar to that of culture medium, negative surface charges on the algae that results in dispersed stable algal suspensions, and the necessity of large volumes of water for cultivation results in low biomass to liquid ratio, which increases the downstream processing cost to about 30–40 % of the total costs of production [3]. Conventional algae harvest technology is generally based on centrifugation, filtration, flocculation, and flotation or a combination of these methods. Apart from the conventional algae harvesting processes, attached microalgae cultivation is another approach to aggregate microalgae on a solid surface during the cell cultivation [4–6], but the process is limited to few algal species. Also, the harvest efficiency of these processes is low due to the difficulty for microalgae cells to aggregate or attach, and they are difficult to scale up. Recently an algal biofilm membrane photobioreactor equipped with solid carriers and a submerged membrane module was developed for attached growth of *Chlorella vulgaris* which has a harvest efficiency of 72.4 % [6]. A laboratory scale rocker system was designed with a polystyrene foam bottom for attachment of a *Chlorella* culture with dairy wastewater as a culture medium for nutrient removal [4]. Research on attached algal growth is limited, but most of the results reported so far have established that the attached culture gave higher yields or comparable lipid content with the suspended culture grown under similar conditions [4]. Attached algal culture systems like algal turf scrubbers are also widely employed to remove nutrients from animal wastewater, in which filamentous benthic algae grow on the surface of solid support [7]. Immobilized algae cells provide (1) more flexibility in the reactor design when compared with conventional suspension systems, (2) accelerated reaction rates due to increased cell density, (3) increased cell wall permeability, (4) no washout of cells, and (5) better operational stability over free-living cells. Bio-flocculation of a non-flocculating microalga (*C. vulgaris, N. oleoabundans*) with another auto-flocculating (*A. falcatus, S. obliquus, T. suecica*) microalga was also evaluated for algae harvesting [8].

Bio-flocculation is similar to activated sludge flocs but this method needs additional sedimentation procedures and separation processes with relatively low recovering efficiency. It was found that the increase in the ratio of the bio-flocculating microalga and the non-flocculating microalga lead to higher sedimentation rates [8]. A bioflocculant from *Paenibacillus* sp AM49 was used to harvest *C. vulgaris*, reaching 83 % harvest efficiency [9], when compared to the 72 % by aluminum sulfate and 78 % produced by polyacrylamide [9]. This method needs an additional bioprocessing step to produce bio-flocculants and may not be an economically viable option. A co-pelletization method was successfully developed in addressing the algae harvesting issue, achieving more than 98 % harvest efficiency using a fungi *Aspergillus niger* [10]. However, this method seems to be limited to pellet forming filamentous fungus and may not have synergistic effect with the required product.

In this present work, we attempt to develop a novel mycoalgae biofilm using a synthetic community which will possibly transform the algae cultivation process in the future for various bioprocessing and bioremediation applications. It is expected that the combined effectiveness of algae and fungi as a biofilm can be a platform technology to be applied in various algae processes, water treatment etc. Even though the mutualistic interactions between algae and non-lichen fungi has been observed earlier [11], this is the first of its kind in developing an artificial lichen type “mycoalgae” biofilm completely attached on a matrix.

## Results and discussion

### Synthetic lichen concept: biofilm formation with algae and fungi

**Fig. 1:**
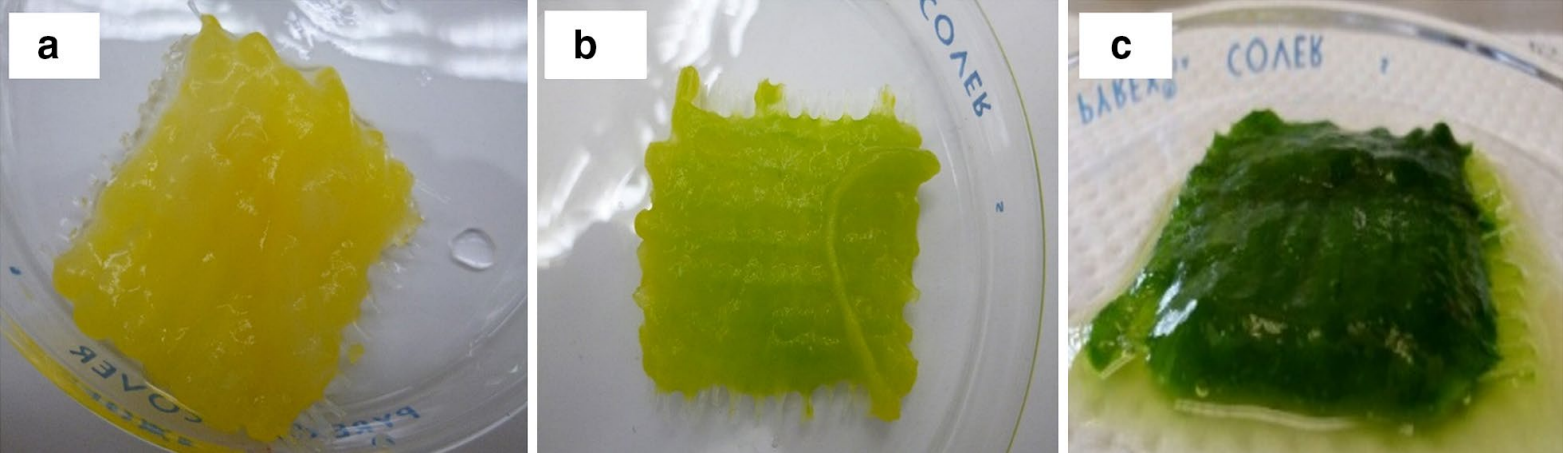
Attached lichen-type mycoalgae biofilm in polypropylene spun-tape yarn composite matrix **a** Biofilm of axenic *Mucor circinelloides* **b** *Mucor circinelloides* and *Chlorella vulgaris* mycoalgae biofilm at initial stages of the biofilm formation (48 h) **c** Mature mycoalgae biofilm at 168 h after complete attachment of the algae

Enabling cells to aggregate during cultivation is a common industrial fermentation technology, widely seen in the fungal conversion process where the microorganisms are filamentous [12]. However, most of the oleaginous microalgae cannot pelletize by themselves or attach to a matrix. A novel eco-friendly algae harvesting technology using the inherent property of pellet formation of fungal cultures was successfully employed to recover algae from the culture medium [10], but this process is limited to the pelletizing fungi and the fungi may also not be synergistic with the product of importance. In the current process, we introduced a supporting matrix to develop a novel microalgae cultivation platform technology using a bio-augment filamentous fungus and microalgae to form a lichen type of biofilm. This type of biofilm will facilitate easy biomass harvesting and a low-cost energy-efficient cultivation technology, which can reach more than 99 % of harvest efficiency of algae from the culture flask. The result shows that certain species of fungi strongly attach to algae to form a composite biofilm containing filamentous fungi and microalgae which attach on a matrix (Table 1). The strain combination *C. vulgaris* and fungi *M. circinelloides* when co-cultured in the presence of an attaching matrix in certain conditions, show that they naturally grow attached on the matrix to form the “mycoalgae biofilm” (Fig. 1). The axenic culture of fungi strain *M. circinelloides* showed complete attachment on the matrix (Fig. 1a) as the cultivation broth remained mostly clear and transparent during the cultivation, while the axenic culture of microalgae *C. vulgaris* remain suspended and there is no attachment of cells on the matrix. In laboratory scale, once the algae is harvested using fungi, the following steps for dewatering is simply managed by placing them on a paper towel without losing algae and can also be achieved by natural air drying. Since, the fungi we intend to use is lipid accumulating species isolated from oil-rich plants we consider that the composite biofilm can be directly used for oil extraction and scaling-up should be relatively simple. Recently, *M. circinelloides* genome has been sequenced, as fungal lipids are gaining more attention as a replacement to plant oils as a feedstock for biodiesel production.

**Table 1:** Comparison of Harvest efficiency (%), concentration ratio of algae axenic culture to algae co-culture, total biomass and percentage biomass composition in co-culture and in axenic (control flask) algae and fungal cultures in *Chlorella vulgaris *(CV) with different fungal species: *Mortierella isabellina* (​MI), *Fusarium equiseti* (A11); *Fusarium lacertarum* (A13); *Nigrospora oryzae* (A16); *Altermaria alternate* (A20); *Fusarium equiseti* (B5); *Mucor hiemalis* (B7) and *Mucor circinelloides* UMN-B34 (MC)

Strain combination	MI-CV	A11-CV	A13-CV	A16-CV	A20-CV	B5-CV	B7-CV	MC-CV
Harvest efficiency (%)	41.15 ± 0.6	82.42 ± 0.47	34.39 ± 4.1	92.90 ± 0.39	99.85 ± 0.03	34.85 ± 8.1	99.89 ± 0.01	99.94 ± 0.02
Concentration ratio of algae axenic cultures to algae in co-culture	6.24	13.15	9.42	27.35	21.81	4.82	3.85	1.52
Total biomass in co-culture (mg/L)	959.0 ± 20.7	655.9 ± 10.1	699.3 ± 115	686.5 ± 17.9	611.6 ± 11.9	1050.5 ± 43.4	689.1 ± 21.8	1243.0 ± 37
% Algae biomass	15.4	10.7	14.08	4.9	6.90	18.3	34.9	48.96
% Fungal biomass	84.6	89.3	85.92	95.1	93.1	81.7	65.2	51.04
Biomass in algae mono-culture (control flask) mg/L	928.1 ± 174	928.1 ± 174	928.1 ± 174	928.1 ± 174	928.1 ± 174	928.1 ± 174	928.1 ± 174	928.1 ± 174
Biomass in fungal mono-culture (Control flask) mg/L	868.5 ± 24	429.0 ± 35	458.0 ± 326	712.5 ± 24	810.50 ± 142	938.5 ± 44	922.0 ± 33	898.50 ± 9.8

### Different matrix for cell attachment and biofilm formation

**Fig. 2:**
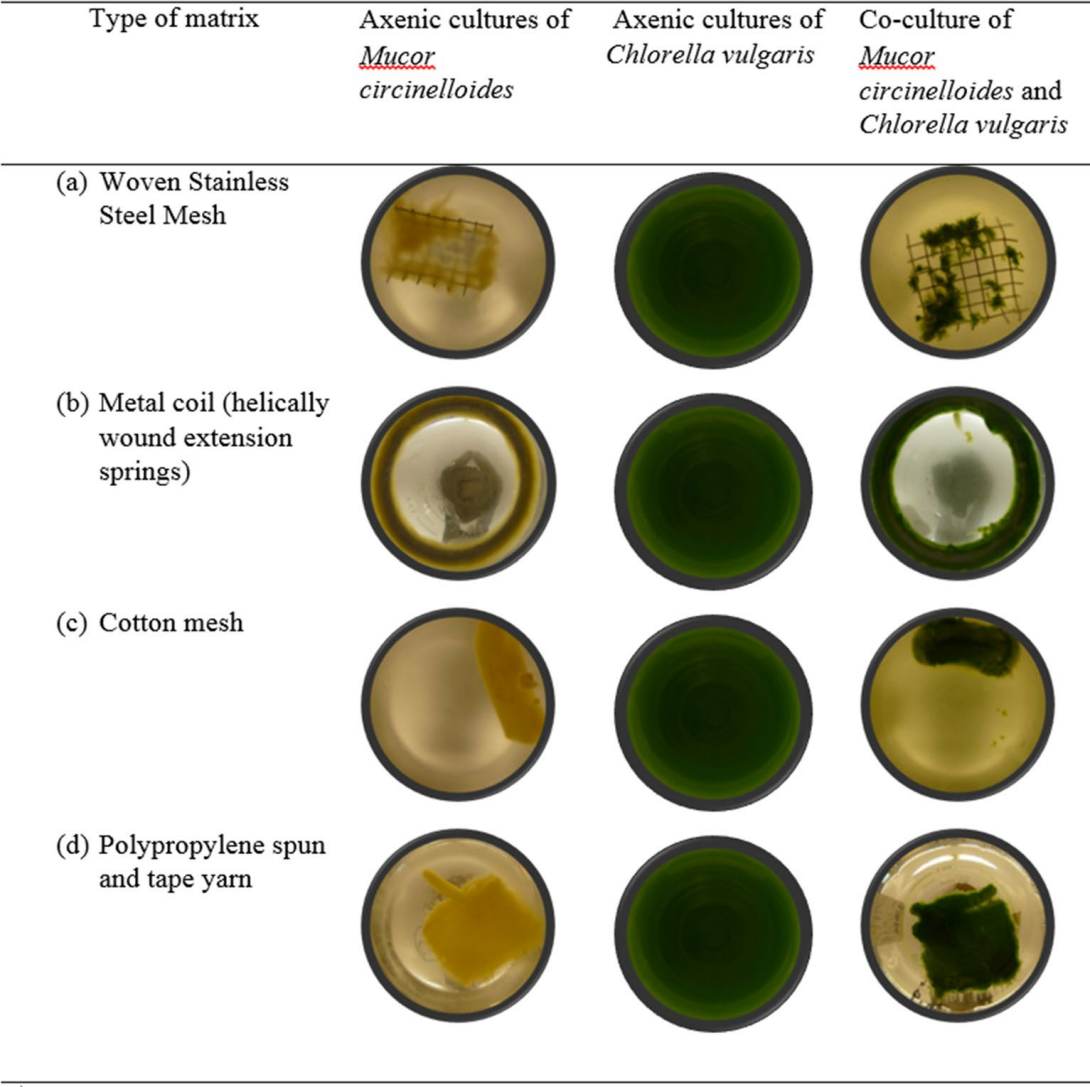
Testing different matrix for attached growth of the Co-culture *Mucor circinelloides* and *Chlorella vulgaris* (Pictures taken from the bottom of the flask)

Selection of the matrix material is an important design factor for scaling up the attached growth bioreactor. Different matrix materials including polymers, polymer-cotton composite, cotton mesh, metal coil (helically wound extension springs) and stainless steel mesh matrix were evaluated for the mycoalgae biofilm development (Fig. 2). The fungal strains exhibit different levels of attachment from no attachment in smooth surfaces either in polymer or metal surface to complete attachment in tape yarn mesh matrix, whereas *C. vulgaris* cells shows poor or no attachment in any of the matrix tested. The stainless steel mesh (Fig. 2a) shows a peculiar type of attachment where the cell growth was predominant in the nodes of the metal mesh, and cells were clustered around that metal intersection point, but in a stainless steel metal coil (Fig. 2b) the cell growth was uniform. The cotton mesh (Fig. 2c) matrix rolled over on itself to form a cylindrical shape at the end of the process, as it lost the stability during the fungal growth but the algae attachment to fungal mycelia was evident in all the experiments. Complete attachment and better cell growth was witnessed in the polypropylene spun and tape yarns woven into a dimensionally stable matrix (Fig. 2d) with the culture solution becoming clear after the attachment, which was chosen for rest of the study based on better cell growth, cost, and reusability.

### Growth of different fungal strains with *Chlorella vulgaris*: percentage algae attachment, biomass production and biofilm composition in the co-culture system

Among the different fungal species tested with the algae *Chlorella vulgaris,* the algae attachment efficiency varies from 34.3 to 99.9 % (Table 1) depending upon the type of fungal strain tested with *Chlorella vulgaris*. The lichen-type biofilm was complete and all the algae cells attached to the *Mucor* sp. with high harvest efficiency, especially with the *M. circinelloides* with high harvest efficiency of 99.94 %. The algae biomass concentration in the control experiments (mono-cultures) was compared with the co-culture concentration; and the ratio of algae in control experiments to the co-culture experiments concentration vary in the range of 1.52–27.35. The lowest attachment efficiency of algae cells was observed in *Fusarium* sp. (34 %) and in *Mortierella isabellina* (41.15 %) but the total cell mass production is not the least in these two cases (Table 1). The lowest algae concentration was found in the *Nigrospora oryzae* which is about 27.3 times less than the axenic algae controls, followed by *Altermaria alternate*.

The harvest efficiency and strain compatibility vary with the strains tested but the subsequent steps for process modification and process condition optimization could yield better results for the required output using the lichen-type biofilms. Most of the fungal species can form a biofilm in the presence of a support material, whereas the conditions for cell pelletization based harvest technology is strain-specific and not all the filamentous fungal strains can form pellets during their growth [10], also the pellet morphology is highly sensitive to the environmental conditions. The total biomass in the co-culture flasks is higher than the individual axenic cultures of algae or fungi for certain co-culture combination, if the algae (*C. vulgaris*) growth is not inhibited by the fungal culture (Table 1) and vice versa depending upon the cell population. The total biomass of co-cultured algae with the fungal strains *Mortierella isabellina, Mucor hiemalis* and *Mucorcircinelloides* was found to be higher than the axenic cultures of fungi or algae (Table 1) tested under the same conditions, which shows that these strain combinations are mutually beneficial or either of the strain benefit from the other for better growth. *Mucor* sp. (*M. circinelloides)* show exceptional affinity for algae, with 1.33 and 1.38 fold increase in total biomass compared to algae and fungi mono-culture control flasks respectively. *Mucor* spp. are a dimorphic fungus which can utilize wide range of carbon sources [5]; and currently being extensively investigated for its high capacity for producing lipids [12, 13], carotenes, enzymes [14], anaerobic and aerobic production of ethanol [15] and as a potential host for heterologous protein production [16], with high biomass production [13]. Further research to elucidate the microbial associations and their mechanisms will enable us to have a better choice of strains for specific application.

Maximum total biomass was found in *M. circinelloides*–*C. vulgaris* combination with biomass concentration of 1243 mg/L (48.96 % algae; 51.04 % fungi), whereas the individual cell concentration of *C. vulgaris* and *M.circinelloides* at the same conditions were 928.1 and 898.50 mg/L respectively. Though the mechanism is not completely understood there is literature showing the mutualistic interactions of different co-cultures and an increase in the total biomass production compared to the axenic cultures [10, 17–19]. In solid surface cultures the fast-growing fungi (*Aspergillus niger*) was found to overgrow and completely inhibit the algae growth, whereas the yeast-like fungi (microcolonial fungi) was found to develop close cell wall contacts and no inhibition of either partner was observed [20]. This report complements the present research results that the non-lichenized fungi *Mucor* sp. has the ability to develop cell wall contacts and eventually produce higher levels of biomass. The enhanced total biomass between algae and fungi can be attributed to the complementary exchange of respiratory gases due to the algae supplementing O_2_ for the fungal biomass at the internal structures of the fungal biomass and receiving CO_2_ from fungi, where in otherwise oxygen mass transfer could be a limiting factor at high cell concentrations of fungal biomass. The carbohydrate and protein rich extracellular polymeric substances produced by both the algae and fungal cells may also have a significant effect on the attachment and further proliferation of both the cells. It was reported that thicker biofilms were formed with the binary population biofilm of *Pseudomonas aeruginosa, Klebsiella pneumoniae*, when compared to individual cells [17]. The comparison of the growth and biochemical composition between mixed cultures and monocultures of algae *Isochrysis galbana* 8701 and the yeast *Ambrosiozyma cicatricose* shows that the specific growth rates and biomass concentration of both species were significantly higher in the mixed culture than in the axenic cultures during the corresponding experimental phases [18].

### Cell surface characterization and Microscopic analysis of the biofilm

The attenuated total reflectance-Fourier transform infrared spectroscopy (ATR-FTIR) of the pure culture biomass and co-cultured biofilm was measured to see the surface functional groups responsible for adhesion between the two species (*C. vulgaris* and *M. circinelloides*). An additional figure file shows the ATR-FTIR spectra of the pure cultures (*Chlorella vulgaris*; *Mucor* sp.) and mycoalgae biofilm (see Additional file 1). The C–O stretching vibration of carbohydrates for the biofilm is slightly higher than the pure cultures which may be due to the algae-fungi interaction. A detailed analysis of the ATR-FTIR spectra and the different regions in the spectra (fatty acid region, protein region, mixed region and polysaccharide region) was discussed in the additional document file (see Additional file 2). Since *Mucor* spp. show dimorphism based on the oxygen availability (takes yeast form under anaerobic conditions), the composition and organization of the cell wall differ greatly in *Mucor* yeasts and hyphae (deposition of new wall polymers is isodiametric in yeasts and apically polarized in hyphae) [21]. FTIR analysis of the samples are predominantly the hyphae structures of the *Mucor* sp. Table 2 gives the molecular cell surface composition of the fungi *Mucor* sp. hyphae and the algae reported in literature [21, 22]. The chemical composition of the cell wall provided in literature varies widely based on the species and culture conditions.

**Table 2:** Comparative analysis of the cell wall composition of the *Mucor* sp. and algae *Chlorella* sp.

Species	Cell wall composition (% dry weight of cell wall)											Ref.
*Mucor* sp.	Chitin	Chitosan	Mannose	Fucose	Galactose	Glucuronic acid	Glucose	Protein	Lipid	Phosphate	Unknown substances	
*M. rouxiia* (hyphae)	9.4	32.7	1.6	3.8	1.6	11.8	0	6.3	7.8	23.3		[20]
*Chlorella vulgaris*	30 (total saccharides)							2.46	15.0		52.54	[21]

The carbohydrate composition varies strongly based on the algae strain and cultivation conditions

**Fig. 3:**
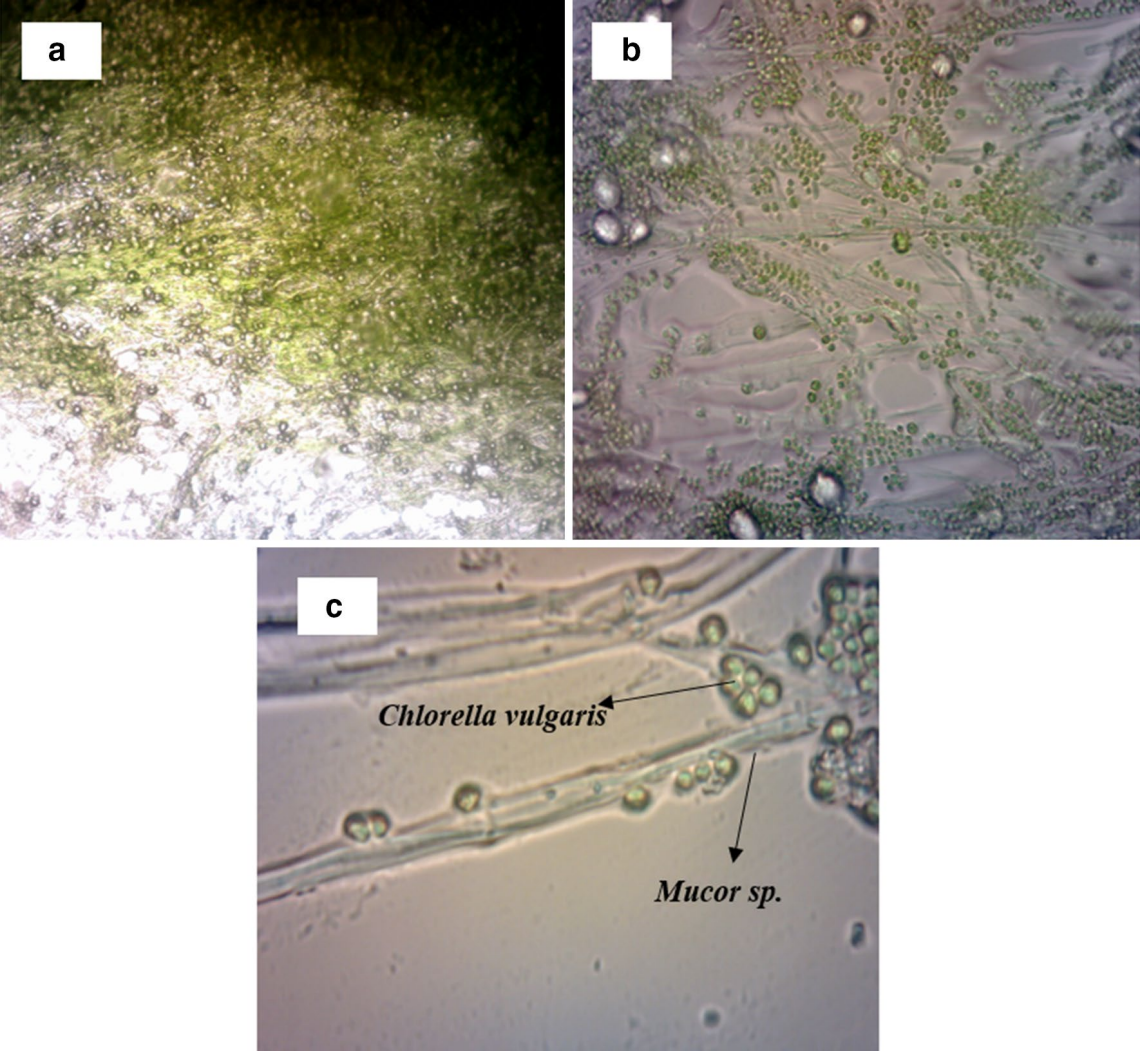
Digital microscopic pictures of the *Mucor circinelloides* and *Chlorella vulgaris* mycoalgae biofilm at different magnification **a** 10×, **b** 40×, **c** 100×

Microscopic image analysis of the co-pelletization process (*C. vulgaris* and *A. niger*), and the biofilm formation (*C. vulgaris* and *M. circinelloides*) (Fig. 3) shows that the green algae cells are entrapped in the fungal mycelia and attached on the fungal cell surface [10]. The zeta potential measurements on the two strains (*C. vulgaris* and *A. niger*), in pellets failed to give any conclusive results of attachment mechanism as the two strains maintained negative zeta potential throughout the process [19], but there was a change in the degree of repulsion and dispersion between these organisms which could have facilitated the attraction between them. In contrary, the zeta potential measured for the fungal strain *A. flavus* and the microalgae revealed that the average zeta potential of micro-algae was−23.7 mV and for *A. flavus* +46.1 mV, which may have contributed to the *A. flavus* ability to capture micro-algae [23].

### Kinetic profile and biomass distribution of the mycoalgae biofilm

**Fig. 4:**
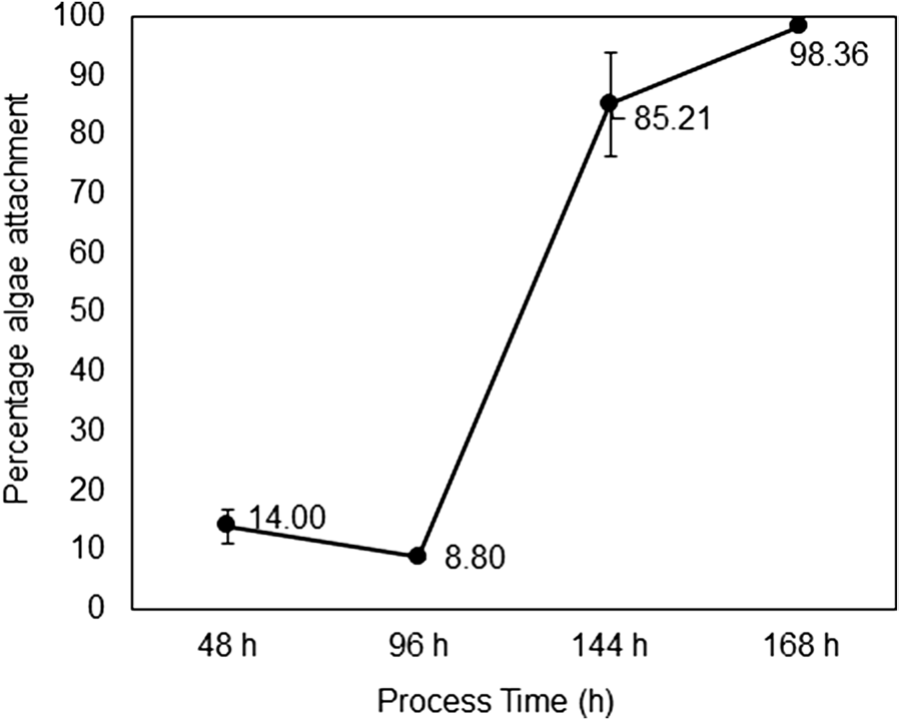
Algae attachment efficiency in the fungal biofilm growth at different process time

**Fig. 5:**
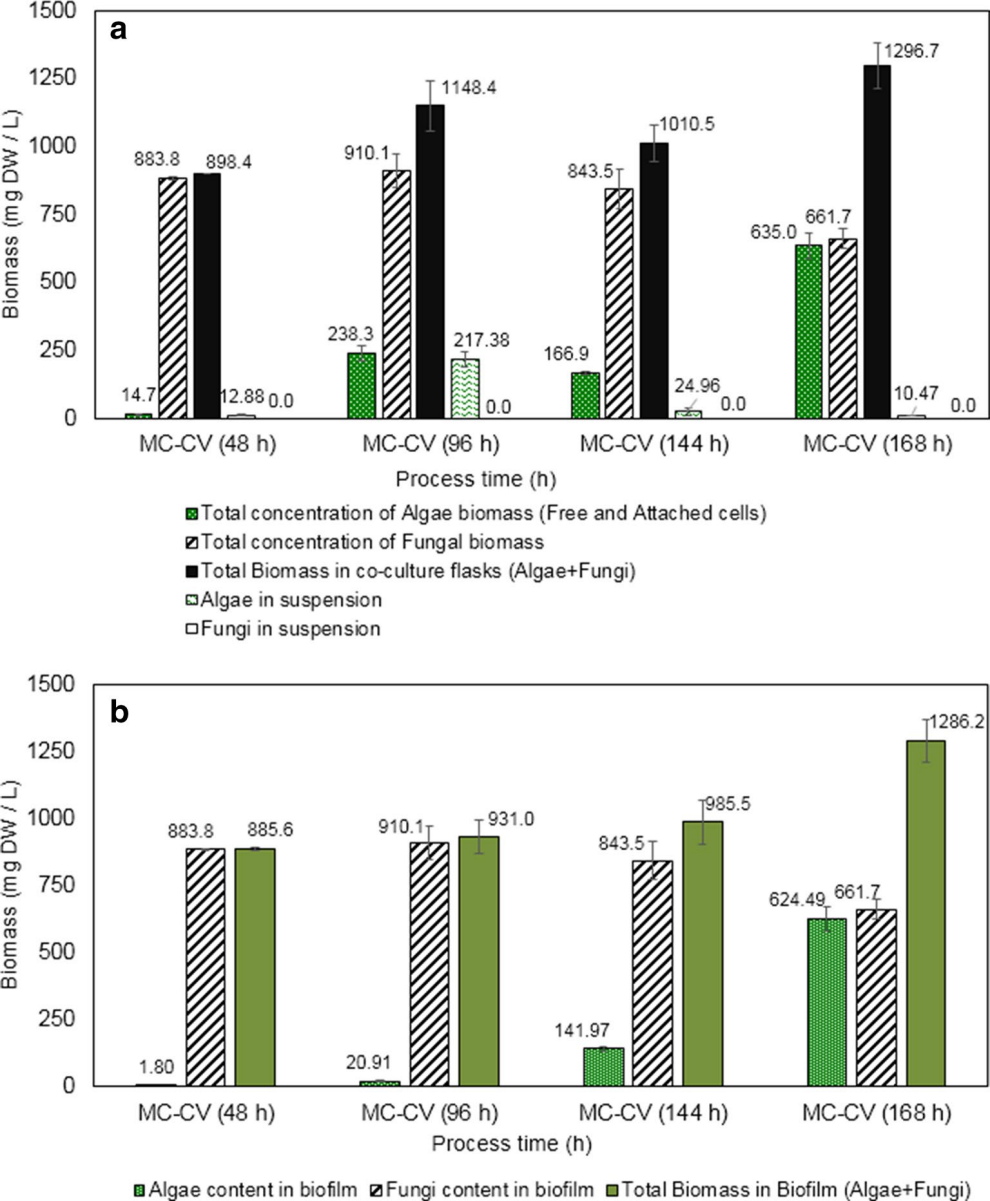
Kinetic profile of the cell cultures of *Mucor* *circinelloides* UMN-B34 with *Chlorella vulgaris* attached on a polymer matrix **a** Total biomass distribution in the co-culture flasks **b** Biomass composition of the mycoalgae biofilm. MC *Mucor circinelloides*; CV *Chlorella vulgaris*

The algae harvest efficiency varies during the process duration (Fig. 4) depending upon the individual cell growth rate and environmental conditions. To understand the growth profile of the individual cells in the biofilm and their nutrient requirements, measurements were made on the total biomass distribution in the co-culture flask, biofilm composition (Fig. 5), glucose and other nutrient concentrations. In co-culture flasks (*C. vulgaris* and *M. circinelloides*), the growth of fungi was predominant during the initial phase (Fig. 5) and the glucose was completely depleted within the initial 48 h. No suspended cell growth was evident in the liquid, so the total biomass was almost the same as the attached biomass until 48 h. The inoculated alga was completely attached to the fungal biomass at 48 h of the process. The low growth rate of the algae during the initial period of the process may also be due to the high accumulation of carbon dioxide, suppressing the growth of algae [24]. After 48 h, algae starts to grow in liquid suspension overcoming the initial inhibition by the fungal cells and metabolites, possibly high CO_2_ release as the growth rate of fungi was more during this period. Since the added carbon (2 g/L) was completely utilized within 48 h, mostly utilized by the fungal cells, it can be presumed that the algae growth after 48 h of the process was predominantly by photoautotrophic mode and partially by the chemicals or exudates released by the fungal biofilm in the liquid. The algae cells in the suspension increases after 48 h and biomass in the matrix also increases gradually. At this stage the attachment efficiency was low due to the high concentration of algae in suspension. At 144 h, all the algae produced become attached to the fungal matrix and the algae attachment efficiency was about 99 % and above. At the end of the process, the total biomass concentration is 1296.7 mg/L (Fig. 5a) and 99.2 % of the biomass is attached as biofilm in the matrix, with the biofilm composition being 48.5 % algae and 51.46 % fungi (Fig. 5b). The culture medium looks clear, which can be used for the next batch of cultures. Preliminary experiments with the recycled water show that the water can be recycled up to three cycles with nutrient addition, without affecting the total biomass production.

The pH of the culture medium gradually increases from 6.4 to 8.3 which may be due to the growth of algae and photosynthesis. The dissolved oxygen concentration of the medium and the pH is usually regulated by the algal photosynthesis and fungal growth; and the net addition or removal of carbon dioxide. Rapidly growing algae remove CO_2_ from the water during photosynthesis, which increases the pH. The constant increase of pH with time after the complete attachment also shows that the algae cells are growing on the fungal surface. Since the initial glucose concentration used was 2 g/L, the organic acid production by fungi was not perceptible, as observed with the gradual rise in the pH. There was a sharp decline in the phosphorous concentration from 35 to 3.36 mg/L within 48 h, which shows the P accumulating capacity of *M. circinelloides* [25]. It was previously established that *M. circinelloides* UMN-B34 can accumulate polyphosphate as luxury uptake for phosphorus storage [25]. This finding indicated that the microalgae cells directly or indirectly utilized polyphosphate accumulated by the fungal cells in conditions in which P was insufficient in the solution to support the further microalgae growth. The cellular phosphorous content of the total biomass was approximately 3.6 % at 48 h, in which the *M. circinelloides* contribution will be major as the algae cell concentration was low at this stage. At the later stages of the process the total phosphorous in the culture medium was found to increase marginally which could be the result of fungal cell lysis or may be due to the release of fungal surface phosphorous due to the algal attachment. The best conditions for developing the lichen type biofilm in this work are given in Table 3.

**Table 3:** Suitable conditions for the lichen biofilm formation with better algae attachment

Parameters	Conditions tested	Suitable conditions for lichen-type biofilm formation
Different fungal strains with *C. vulgaris*	*Mucor* *circinelloides* UMN-B34; *Fusarium equiseti* (A11); *Fusarium lacertarum* (A13); *Nigrospora oryzae* (A16); *Altermaria alternate* (A20); *Fusarium equiseti* (B5); *Mucor hiemalis* (B7) *and Mortierella isabellina* (MI)	*Mucor* sp. UMN B34
Matrix type	Cotton/cotton-polypropylene/metal matrices	Polypropylene-Yarn
Duration of the process	0–180 h	After 160 h > 98 % algae attachment

### Attached growth and possible mechanism of Mycoalgae biofilm formation

The cell surfaces in biofilms are often considered soft and covered with highly hydrated flexible macromolecules [26], usually the extracellular polymeric substance consists of proteins, lipids, and lipopolysaccharides facilitating adhesion between cells and cell to surfaces. Algae and fungal cells are capable of producing surface macromolecules, and the macromolecular interactions in this case tested are often polymer bridging. But in some cases, the macromolecules can also be repulsive depending on the coverage degree, macromolecule characteristics, and type of solvent [26]. Formation of mycoalgae biofilm on a matrix is a sequential process involving (1) adherence of fungal spores [27] or germinating fungal spores and algae onto a matrix, (2) proliferation of yeast-type cells over the surface as the *Mucor* sp. is dimorphic, and (3) induction of hyphal formation [28]; together with the attachment of algae on the fungal surface. It is also understood that the spores of the fungal species have crystalline-like rodlet layers preventing aggregation within themselves and with other strains, but the macromolecules on the growing hyphae would promote bridging interactions between germinating spores and essentially between other species [26]. The surface protein, especially Ca^2+^ dependent lectin-carbohydrate attachment [29] or protein–protein interaction may play a role in the strong binding of algae and fungi. Ca^2+^ is also found to act as a link between negatively charged cell surface and extracellular DNA, enabling cell–cell attraction through electrostatic interactions for biofilm formation in bacteria [30]. The reason for the weak binding of lectin-carbohydrate to form stable cell aggregates and multifold increases in the shear resistance is still not well-understood but it was found that the magnitude of cell–cell adhesion forces increased with contact time [31].

Biofilm heterogeneity and the position of the cells in the microbial biofilm determine the transport phenomena in biofilms [32] which can be witnessed using advanced microscopic methods. The attachment of algae and fungal can also be visualized using high magnification microscopic pictures (Fig. 3). It is clear from the digital microscope picture that the algae cells are firmly attached to the fungal surface, which might be due to the extracellular substances released from both the cells or by lectin-like interaction, which is yet to be established. There are few clusters of algae at certain places where the fugal mycelia branch and form a nest or V-shaped structure, and it can be clearly seen that some algae are interlocked between the filamentous fungi, which will certainly have more shear resistance than the surface attached algae cells.

**Fig. 6:**
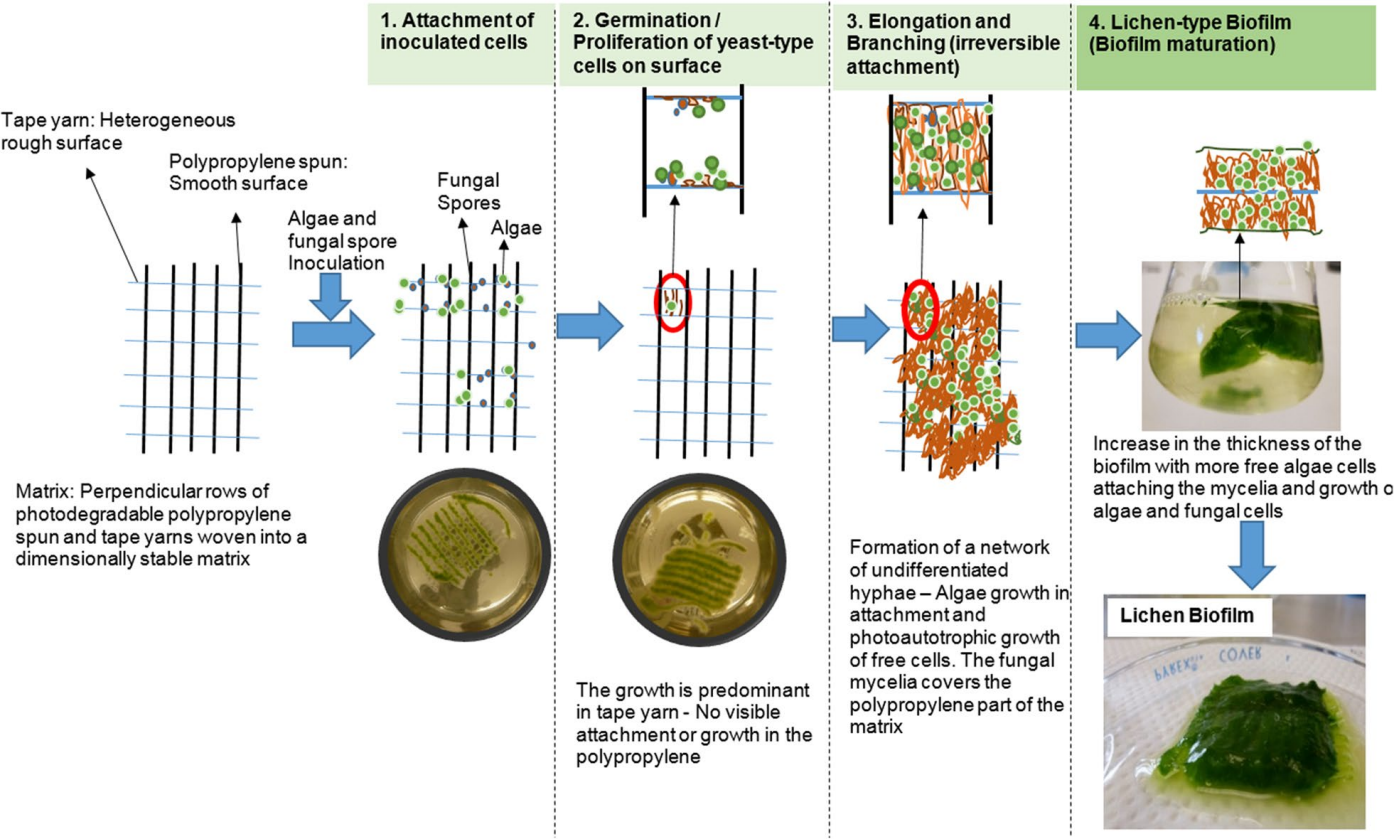
Possible mechanism and stages of algae–fungal cell attachment and proliferation of the mycoalgae biofilm

Based on the visual observation and literature analysis, four important steps (Fig. 6) in the lichen-type biofilm formation are most likely in a polypropylene spun and tape matrix as discussed below: (1) Preferential attachment of strains: The highly hydrophobic fungal spores attached preferentially to the tape yarn of the matrix within a few hours, whereas the algae cells attach to the fungal cells just after spore germination. The polypropylene component of the matrix only gives mechanical stability. The characteristics of the matrix may have a significant effect on the rate and extent of attachment by microorganisms. The rougher and more hydrophobic materials will develop biofilms more rapidly [33]. (2) Germination of fungal spores: The attached fungal spores propagate along with the attached algae cells to form micro-colonies. It is not clear, what type of dimorphism is predominate in the initial stages of the fungal growth. But based on the literature, the yeast-type could proliferate over the surface of the matrix initially [28]. The alga cells were almost covered by the fungal cells (Fig. 1b) and the growth of the algae is not predominat at this stage. (3) Elongation and branching (induction of hyphae formation): The fungal cells fill the space in-between the perpendicular rows of polypropylene spun and tape yarn by the formation of a network of undifferentiated hyphae. It was shown that the *C. albicans* biofilms are comprised primarily of yeast-form and hyphal cells, both of which are required for biofilm formation [28]. Since the *M. circinelloides* strain is dimorphic, the biofilm of *M. circinelloides* could have both the yeast-form and hyphae. The algae grow in the liquid, mostly autotrophically as the carbon source is predominantly consumed by growing fugal cells. (4) Mature mycoalgae biofilm: The thickness of the biofilm increases and more free algae cells attach to the fungal mycelia, for which the reason is not clear. Extracellular polymeric substances accumulate as the biofilm matures and may contribute to the cell cohesion or the surface energetics could contribute to cell–cell attraction.

Physical examination of the mature biofilm shows that the algae attachment and growth was predominant in biofilm surface exposed to the light, when compared to the algae cells at the bottom of the biofilm, which shows that the algae prefer to attach on the light exposed surface and continue propagating on the fungal surface after complete attachment. A suitable reactor design with the entire fungal surface exposed to the light source could enhance the algae attachment and growth. The key parameter for the success of this process is the selection of suitable strain combination which should contribute or have synergetic effect on the product of interest; otherwise the fungi may have a negative effect on the productivity and yield of the product. The concept of nutrient exchanging mycoalgae biofilm can be developed for other bio-production and bioremediation applications with suitable cell combinations. This composite biofilm will also have the capacity to remove pollutants in a wide concentration range, and to possibly recycle valuable and non-renewable resources. The future research focus on cell surface properties and the molecular mechanism of understanding the attachment should provide detailed insight on cell to cell and cross species interaction and economical industrial applications of algae-fungi lichen like biofilms including biofuels, bio-products, and bioenergy.

## Conclusions

Studies carried out for the identification of a suitable strain combination and condition for biofilm formation revealed that few fungal strains can grow together with the model strain *C. vulgaris* to form a lichen-type mycoalgae biofilm. The algae attachment efficiency of 99.94 % was observed with higher total biomass in co-cultures than in the axenic cultures. A suitable matrix for the attachment was identified and the algae growth was evident on the fungal surface at carbon limited conditions which is commendable for the bioremediation applications. The pH measurement and the microscopic observation show that the algae seems to colonize and continue to grow as attached cells on the fungal surface. These findings show that the mycoalgae biofilm has potential applications in industrial bioprocessing systems and also for varieties of environmental bioremediation applications, especially involving algae, where the separation of algae from the medium is the most expensive and energy intensive process.

## Methods

### Microorganisms

*Chlorella vulgaris* 2714, unicellular green microalgae was selected as the model algae strain for the mycoalgae biofilm formation. The strain was obtained from the Culture Collection of Algae at the University of Texas (UTEX). The fungal strains used in this work for co-culturing with algae were isolates from the soybean, soybean hull, and soil samples surrounding the soybean roots collected from croplands in Rosemount, MN [34]. The fungal strains used in our study were *Mucorcircinelloides* UMN-B34; *Fusarium equiseti* (A11); *Fusarium lacertarum* (A13); *Nigrospora oryzae* (A16); *Altermaria alternate* (A20); *Fusarium equiseti* (B5); *Mucor hiemalis* (B7) and *Mortierella isabellina* (MI).

### Culture medium and maintenance

The microalgae was cultivated and maintained in medium (A) that contained (g L^−1^): Glucose 2, KNO_3_ 1, KH_2_PO_4_ 0.075, K_2_HPO_4_ 0.1, MgSO_4_.2H_2_O 0.5, Ca(NO_3_)_2_.4H_2_O 0.0625, FeSO_4_.7H_2_O 0.01, Yeast extract 0.5, and Trace metal solution 1 ml L^−1^. The trace metal solution contained (mg L^−1^): H_3_BO_3_ 2.86, Na_2_MoO_4_.2H_2_O 0.39, ZnSO_4_.7H_2_O 0.22, MnCl_2_.4H_2_O 1.81, CuSO_4_.5H_2_O 0.079, and Cu(NO_3_)_2_.6H_2_O 0.049. The stock cultures of the algae were maintained in the agar slants at 25–27 °C (Media A + 1.5 % Agar) under white fluorescent light illumination and periodically revived. The fungal spores were preserved in 60 % glycerol solution at −80 °C. The glycerol stocks were aseptically streaked on potato-dextrose agar plates and incubated at 37 °C. The spore solution was prepared using sterile water and stored at 4 °C for inoculation. For inoculating the algae cells and fungal spores, the cell count of algae and fungal spores was done using a 0.1 mm deep Neubauer improved haemocytometer (Hausser Scientific, USA) under microscope (National DC5-163 digital using 40× magnitude).

### Erlenmeyer shake flask cultures

The experiments were conducted in 250 mL Erlenmeyer flasks with 100 mL of the culture medium and a submerged supporting matrix for biofilm formation. The medium was adjusted to an initial pH of 6.8 using 2 mol/L HCl or 1 mol/L NaOH (pH meter Oakton, SN 153,400, Malaysia), and heat sterilized (250 °F, 15 psi for 20 min) along with the matrix. The culture medium was inoculated with the co-cultures of fungal spores and algae cells at a ratio of 1:300 (initial algae count: 2.50 × 10^9^ cells) unless otherwise specified and incubated in an orbital shaker at 150 rpm and 26 °C in the presence of light (light intensity of continuous illumination was set to 100 µmol/s/m^2^) for the entire cultivation period of about 8 days. Aliquots of samples from the cultivation broth were withdrawn at regular time intervals for glucose analysis and cell counts of suspended algae without much change in the culture volume to maintain constant oxygen transfer. The suspended cells were separated from the medium by centrifugation for 15 min at 4 °C and 5030*g* and filtered through a 0.45 µm filter for residual nutrient analysis. Glucose concentration was estimated using DNS method [35]. The total phosphorous content in the culture liquid was measured using Hach analysis kits (Hach Company, Loveland, CO), following the standard protocol described in the kit manual. Control experiments with axenic cultures were also performed under the same conditions tested for the co-culture experiments.

### Testing the process conditions

The possibility of co-existence of *Chlorella vulgaris* with various fungal cultures listed in experimental methods was tested initially to identify the best strain combination for high harvest efficiency. The harvest efficiency and biomass distribution was analyzed after 7 days of inoculation. Different matrices (polymers, polymer-cotton composite, cotton mesh, metal coil- helically wound extension springs and stainless steel mesh matrix) were tested for better attachment of fungal cells and the mycoalgae biofilm formation using the best strain combination. The cellular growth kinetics, pH variations and the harvest efficiency at different time intervals was observed under different conditions.

### Cell harvest

After the completion of biofilm formation or observing the complete attachment of algae in approximately 8 days of culture, the mycoalgae biofilm was removed from the flask and analyzed for biomass distribution. Weight ratios of the wet to dry samples were measured to calculate the amount of dry biomass taken for chlorophyll analysis. Pictures were taken at different stages of the cell culture and biofilm formation with a digital camera (DSC-T20, Sony).

### Biomass distribution and harvest efficiency

The microalgae cell numbers in the supernatant were measured after diluting the supernatant multiple times until the cell numbers can be counted under microscope. Algal biomass in the biofilm samples was determined indirectly by measuring chlorophyll-a (Chl-a) concentration and determining the algal biomass using a standard chart of Chl-a concentration and dry biomass. Chl-a concentration is determined spectrophotometrically (Shimadzu UV spectrophotometer, UV-1800, Torrance, CA, USA) by homogenizing and extracting with methanol solution (90 % v/v) at 650 and 665 nm [36]. The chlorophyll a and algal biomass was correlated using the standard equation: Dry algae mass (mg) = 8.0372 (A_650nm_) [R^2^ = 0.99]. The fungal biomass in the biofilm was determined by the difference from the total dry weight of the biomass and the algae biomass in the matrix. The percentage of microalgae harvesting efficiency is the amount of microalgae biomass attached to the polymer over the total algae biomass produced. The total biomass in the flask cultures were determined by gravimetric method (oven-dried overnight at 105 °C), with the weight of the matrix excluded. The mycoalgae biofilm was viewed using a digital microscope (National DC5-163) connected to a computer using Motic Images plus 2.0 software. All values given here in the manuscript were means of triplicate determinations.

### ATR-FTIR Measurements of the biomass

The biofilm samples and algae biomass after complete growth were removed from the matrix or centrifuged (for pure algae samples). The samples were washed three times and aliquot of samples were freeze dried for FTIR analysis (Thermo Scientific Nicolet iS50 FT-IR spectrometer with a built-in diamond attenuated total reflection (ATR) with deuterated l-alanine doped triglycine sulfate (DLaTGS) detector). A total of 100 scans at a resolution of 4 cm^−1^ were averaged for each sample, and the strong spectral contribution of water in the wet paste sample was removed from the ATR-FTIR spectrum by subtracting the spectrum of the corresponding supernatant solutions. The spectra were analyzed using OMNIC software (Nicolet Instrument Corporation, USA).

## Additional files

10.1186/s13068-016-0533-y ATR-FTIR spectra of the pure cultures (*Chlorella vulgaris*; *Mucor sp*.) and mycoalgae biofilm. Region 1 is the fatty acid region, Region 2 is the protein region, Region 3 is mixed region and Region 4 is polysaccharide region.
10.1186/s13068-016-0533-y Attenuated total reflectance—Fourier transform infrared spectroscopy of the Biofilm and pure cultures of algae and fungi.

## Abbreviations

Chl-a: chlorophyll-a
MC: *Mucor**circinelloides* UMN-B34
CV: *Chlorella vulgaris*
ATR-FTIR: attenuated total reflectance-Fourier transform infrared spectroscopy
EPS: extracellular polymeric substances
