# Supplementary figures and images for: Mycoalgae biofilm: development of a novel platform technology using algae and fungal cultures
Source: Biotechnol Biofuels. 2016 May 31;9:112. doi: 10.1186/s13068-016-0533-y (PMC4886447; doi:10.1186/s13068-016-0533-y)

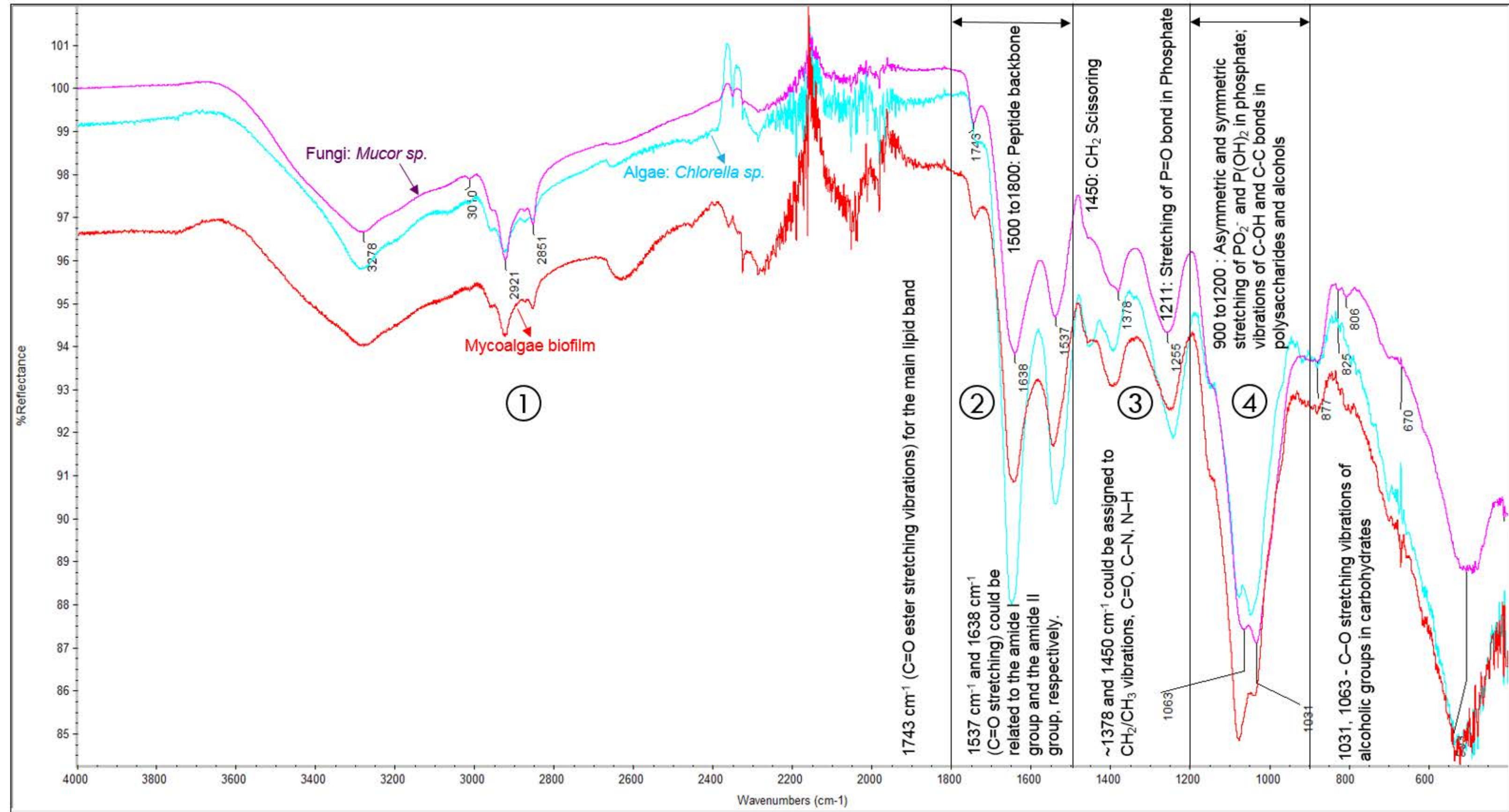

Supplement: Supplementary file 1 — 10.1186/s13068-016-0533-y ATR-FTIR spectra of the pure cultures (Chlorella vulgaris; Mucor sp.) and mycoalgae biofilm. Region 1 is the fatty acid region, Region 2 is the protein region, Region 3 is mixed region and Region 4 is polysaccharide region. [file 13068_2016_533_MOESM1_ESM.pdf]
