# Supplementary material for: Mycoalgae biofilm: development of a novel platform technology using algae and fungal cultures
Source: Biotechnol Biofuels. 2016 May 31;9:112. doi: 10.1186/s13068-016-0533-y (PMC4886447; doi:10.1186/s13068-016-0533-y)
Supplement: Supplementary file 2 — 10.1186/s13068-016-0533-y Attenuated total reflectance—Fourier transform infrared spectroscopy of the Biofilm and pure cultures of algae and fungi. [file 13068_2016_533_MOESM2_ESM.docx]

**Mycoalgae biofilm: Development of a novel platform technology using algae and fungal cultures**

**Aravindan Rajendran, Bo Hu***

Department of Bioproducts and Biosystems Engineering,

University of Minnesota,

1390 Eckles Ave, Saint Paul, MN, 55108-6005

Aravindan Rajendran

University of Minnesota, Department of Bioproducts and Biosystems Engineering

Room: 219 BioAgEng, 1390 Eckles Ave.

St. Paul, MN 55108-6005, United States of America

Tel.: 612-624-4915

Email: arajendr@umn.edu

* Correspondence to:

**Bo Hu**

University of Minnesota, Department of Bioproducts and Biosystems Engineering.

Room: 315, 1390 Eckles Ave, Saint Paul, MN, 55108-6005, United States of America

Tel.: 612-625-4215; Fax: 612-624-3005.

Email: bhu@umn.edu, Website:www.bbe.umn.edu

**Additional file 2: Supplementary Document S2.doc**

**Attenuated total reflectance - Fourier transform infrared spectroscopy of the Biofilm and pure cultures of algae and fungi**

The characterization of surface components is the key to understanding the mechanisms of surface-based phenomena, including cell-cell attraction as well as cell-matrix attraction. Cell attachment is a complex process regulated by diverse characteristics of the growth medium, substratum, and cell surface [[1](#_ENREF_1)], where microorganisms irreversibly attach and grow on a surface producing extracellular polymers that facilitate attachment. The electrostatic repulsion or attraction followed by the hydrophobic interactions [[2](#_ENREF_2)] influence the initial attachment and matrix formation, resulting in an alteration in the expression and secretion of chemical factors such as quorum sensing molecules [[3](#_ENREF_3)].

The attenuated total reflectance - Fourier transform infrared spectroscopy (ATR-FTIR) spectrum (supplementary file, Fig. S1) of the pure culture biomass and co-cultured biofilm was measured to see the surface functional groups responsible for adhesion between the two species (*C. vulgaris* and *M. circinelloides*). ATR-FTIR spectroscopy provides molecular-scale information on both organic and inorganic constituents including close proximity investigation of dipolar functional groups involved in cell-cell and cell-surface adhesion [[4](#_ENREF_4)]. It was demonstrated that the ATR-FTIR spectra of bacterial suspensions are likely to reflect only functional groups of cell walls and not intracellular ones [[5](#_ENREF_5)]. The functional groups of microbial surfaces (carboxyl, amide, phosphate, hydroxyl, and carbohydrate related moieties) especially the anionic and cationic groups and hydrophobic properties of cell wall have an effect on flocculation and adsorption properties of the cells [[6](#_ENREF_6)].

The spectral analysis shows that bands between 900 to 1200 cm^-1^ and 1500 to 1800 cm^-1^ are high intensity regions for all three samples tested (pure cultures of algae, fungi, and mycoalgae biofilm) and also has variations between the samples even though other regions are relatively similar. Since region 2 and 4 in Fig. S1 are mainly correlated with the formation of extracellular polymeric substances (EPS), they might represent the physical and chemical properties of the EPS pure cultures vs the co-culture biofilm. The C-O stretching vibration of carbohydrates for the biofilm is slightly higher than the pure cultures which may be due to the algae-fungi interaction. The evidence for mutualistic interactions between rock inhabiting fungi and lichen photobionts was observed in transmission electron microscopy images. There was confirmed close wall-to-wall contacts as well as mucilage production around the contact zone between the algae and fungi [[7](#_ENREF_7)]. The intensity of the amide II in the mycoalgae biofilm is relatively low compared to the pure algae culture but closely resembles the pure fungal culture samples. It is also possible that there might be nutrient transfer between species as the fungi *M. circinelloides* is highly versatile in nutrient utilization which shows differences in the intensity of algae surface proteins. A report on oleaginous genome-scale models has revealed that *Mucor* spp*.* possesses a higher number of genes involved in carbohydrate, amino acid, and lipid metabolism [[8](#_ENREF_8)] and can utilize diverse substrates effectively.

The spectral region from 900 to 1200 cm^-1^ (Fig S1 Region 4) was dominated by the stretching (C–O–C) vibrations of polysaccharides (glycogen) [[4](#_ENREF_4)]. For all three samples, a double peak was observed in the spectral region at 1031 and 1063 cm^-1^ and was probably caused by C–O stretching vibrations of alcoholic groups in carbohydrates, a main component of algae [[9](#_ENREF_9)] and fungi cell walls. For the two species, the spectra were dominated by the 1500–1800 cm^-1^ region (Region 2) due to the absorbance of the peptide backbone. In the spectral region varying from 1200 to 1700 cm^-1^, two exclusive absorbance at approximately 1537 cm^-1^ and 1638 cm^-1^ (C=O stretching) could be related to the amide I group and the amide II group, respectively. The common peaks at ~1378 (Region 3) and 1450 cm^-1^ could be assigned to CH_2_/CH_3_ vibrations, C=O, C–N, N–H, mainly in protein structures which represented a significant part of the cell walls. The peak at 1743 cm^-1^ (C=O ester stretching vibrations) is the main lipid band which could be derived from the ester linkage of the fatty acids (aliphatic monocarboxylic acids). This absorbance at 1743 cm^-1^ can be explained by the outer characteristics of the cell membrane being largely composed of lipopolysaccharide (LPS), which consists of a lipid and a polysaccharide joined by a covalent bond. Region 1 (Fig. S1) explains the fatty acid with 2851 and 2921 cm^-1^ being CH_2_ symmetric stretch and CH_2_ asymmetric stretch.

**References**

1. Donlan RM. Biofilms: Microbial life on surfaces. Emerging Infect Dis. 2002;8(9):881-90.

2. van Oss CJ. Long-range and short-range mechanisms of hydrophobic attraction and hydrophilic repulsion in specific and aspecific interactions. J Mol Recognit. 2003;16(4):177-90. doi:10.1002/jmr.618.

3. Renner LD, Weibel DB. Physicochemical regulation of biofilm formation. MRS Bull. 2011;36(5):347-55. doi:10.1557/mrs.2011.65.

4. Sukenik A, Bilanovic D, Shelef G. Flocculation of Microalgae in Brackish and Sea Waters. Biomass. 1988;15(3):187-99. doi:Doi 10.1016/0144-4565(88)90084-4.

5. Bilanovic D, Shelef G, Sukenik A. Flocculation of Microalgae with Cationic Polymers - Effects of Medium Salinity. Biomass. 1988;17(1):65-76. doi:Doi 10.1016/0144-4565(88)90071-6.

6. Rijnaarts HHM, Norde W, Bouwer EJ, Lyklema J, Zehnder AJB. Bacterial deposition in porous media: Effects of cell-coating, substratum hydrophobicity, and electrolyte concentration. Environ Sci Technol. 1996;30(10):2877-83. doi:Doi 10.1021/Es9605984.

7. Bilanovic D, Andargatchew A, Kroeger T, Shelef G. Freshwater and marine microalgae sequestering of CO_2_ at different C and N concentrations - Response surface methodology analysis. Energy Convers Manage. 2009;50(2):262-7. doi:10.1016/j.enconman.2008.09.024.

8. Vongsangnak W, Klanchui A, Tawornsamretkit I, Tatiyaborwornchai W, Laoteng K, Meechai A. Genome-scale metabolic modeling of *Mucor circinelloides* and comparative analysis with other oleaginous species. Gene. 2016. doi:S0378-1119(16)30095-6 [pii]10.1016/j.gene. 2016.02.028.

9. Aboshady AM, Mohamed YA, Lasheen T. Chemical-Composition of the Cell-Wall in Some Green-Algae Species. Biol Plant. 1993;35(4):629-32. doi:Doi 10.1007/Bf02928041.
